# Supplementary material for: Glypican-3 (GPC3) is associated with MCPyV-negative status and impaired outcome in Merkel cell carcinoma
Source: Oncotarget. 2022 Aug 3;13:960–7. doi: 10.18632/oncotarget.28260 (PMC9348696; doi:10.18632/oncotarget.28260)
Supplement: Supplementary file 1 [file oncotarget-13-28260-s001.pdf]

Glypican-3 (GPC3) is associated with MCPyV-negative status and impaired outcome in merkel cell carcinoma

SUPPLEMENTARY MATERIALS

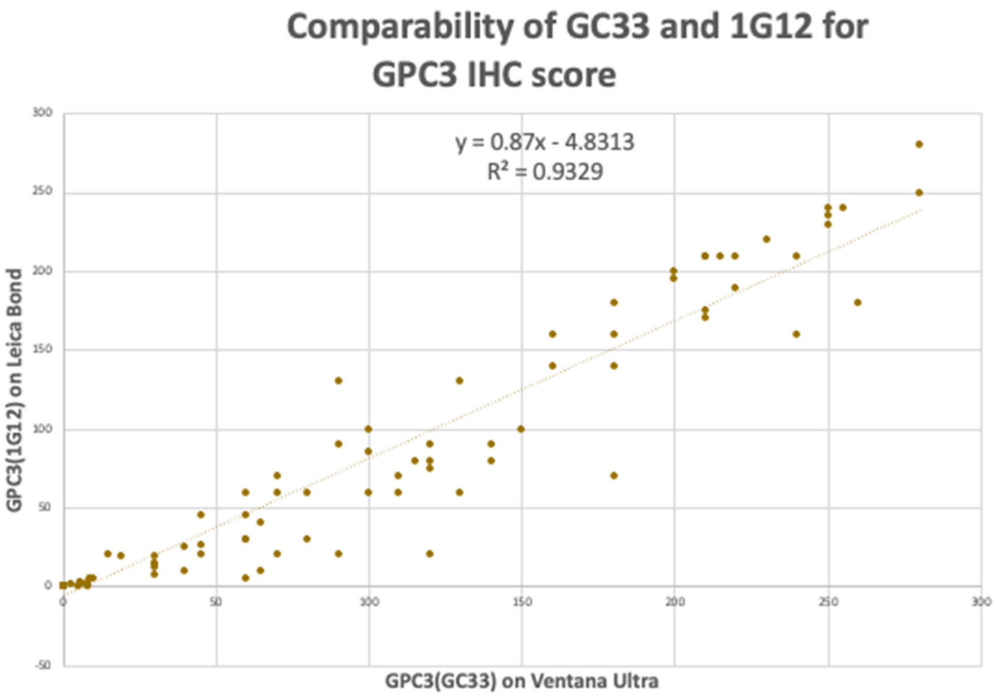

Supplementary Figure 1: Comparisons between the 2 IHC assays. Legend: x-axis is GC33, y-axis is 1G12, best fit lit is plotted with an r^2 co-efficient of 0.9329.

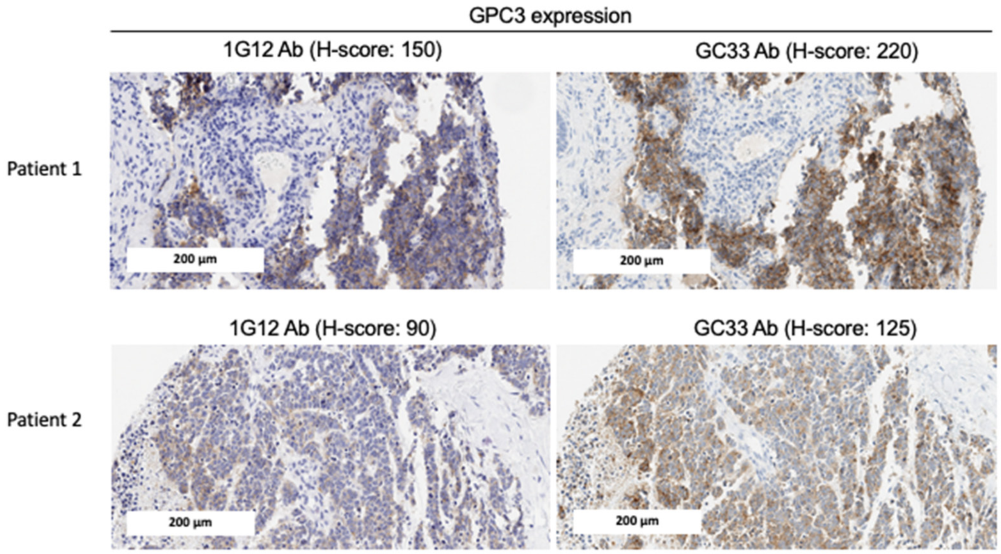

Supplementary Figure 2: Representative images of 2 IHC assays. GPC3 IHC scoring of MCC tumor samples from two patients using 1G12 and GC33 antibodies, respectively. The scale bar represents 200 µm.

Supplementary Table 1: Accuracy, sensitivity, specificity and precision of GC33 staining for GPC3 compared to comparator 1G12 staining

		GPC3 (1G12) Reference IHC		
		**Expected GPC3 Positive**	**Expected GPC3 Negative**	**Total**
**GPC3 (GC33) IHC Staining**	**Observed GPC3 Positive**	73 (True Positives)	8 (False Positives)	48% (81/169)
	**Observed GPC3 Negative**	0 (False Negatives)	88 (True Negatives)	52% (88/169)
	**Total**	43% (73/169)	57% (96/169)	100% (169/169)
